# Editorial: Metabolic architecture of developing seeds and grains

**DOI:** 10.3389/fpls.2023.1258353

**Published:** 2023-08-08

**Authors:** Rachel Amir, Enrique Martínez-Force, Jianxin Shi, Ana Paula Alonso

**Affiliations:** ^1^ Department of Plant Sciences, Galilee Research Institute (MIGAL), Kiryat Shmona, Israel; ^2^ Department of Biotechnology, Tel-Hai College, Upper Galilee, Israel; ^3^ Department of Biochemistry and Molecular Biology of Plant Products, Instituto de la GRASA-CSIC, Seville, Spain; ^4^ Joint International Research Laboratory of Metabolic and Developmental Sciences, School of Life Sciences and Biotechnology, Yazhou Bay Institute of Deepsea Sci-Tech, Shanghai Jiao Tong University, Shanghai, China; ^5^ BioDiscovery Institute and Department of Biological Sciences, University of North Texas, Denton, TX, United States

**Keywords:** seeds, grains, metabolism, development, lipids, fatty acid, amino acids

During development, seeds and grains synthesize and store many valuable sink metabolites using source metabolites imported from vegetative tissues. Related to the genus and species of the specific plants, seeds undergo a specific endogenous metabolic program to fill and store their reserve, mainly biopolymers of carbohydrates, lipids, and proteins (Fait et al., 2006; Baud et al., 2008; Hu et al., 2016; Amir et al., 2018; Moreno Curtidor et al., 2020). These sink metabolites play a vital role in subsequent seed embryogenesis, maturation, desiccation, and germination. In addition to the function of these storage biopolymers during seeds development and germination, these storage compounds are important for: (i) Human nutrition and health due to their high values, including essential fatty acids (FAs) and essential amino acids; (ii) Animal feeding; (iii) Industrial raw materials; and (iv) Renewable biofuels and biodiesels.

Understanding metabolic and developmental control of seed/grain filling of these storage biopolymers is relevant not only to the seed/grain yield and quality, but also to the improvement of seed/grain production through breeding and genetic manipulation. Although most monomers required to form these storage compounds are mostly transferred from the source vegetative tissues (Baud et al., 2008; Hu et al., 2016; Amir et al., 2018; Hu et al., 2020), some of these monomers are synthesized from other metabolites in seeds during development (Baud et al., 2008). The complexity of seed/grain metabolism highlights the metabolic potential of enhancing the storage components and changing their profiles for different nutritional and biotechnological uses.

Lipids play many roles in plant development including seed development as energy reserves, membrane components, protective and structural barriers, and signalling molecules. Among the different lipids detected in the seeds, triacylglycerols (TGs) are a major energy reserve that accumulates during seed development (Durrett et al., 2008; Dyer et al., 2008; Chen et al., 2023; Hong et al., 2023). The importance of lipids in seed/grain development was highly reflected in this Research Topic, as five out of the six publications are related to lipids and oil accumulation.

The seeds of the *Brassicaceae* family are known to produce high levels of oil. The study of Johnston et al., using an integrated comparative metabolomics and transcriptomic analysis, showed the complexity of the biochemical pathways involved in the synthesis of FAs and of lipid packaging in seeds of pennycress (*Thlaspi arvense* L.). The study revealed that the accumulation of long-chain unsaturated FAs requires more carbon flow through malate and pentose phosphates, production of carbon precursors via threonine aldolase activity, alteration of sugar incorporation into cell wall components, and alteration of expression-level of genes associated with ubiquitination and lipid droplet organization. The insights obtained from this study can lead to producing oil in a more efficient way for biofuel and other oil industries.

Lipid synthesis in seeds is a complex system requiring precise and rigorous regulatory mechanisms. One of the master regulators of this process is the transcription factor *WRINKLED1* that controls the synthesis of TGs during seed development. Kuczynski et al. searched for novel targets of this factor in addition to those that directly control the expression of many genes in FAs biosynthetic pathway and a few genes in glycolytic pathway. Using the phylogenetic approach, the researchers searched for genes that have the DNA-binding consensus in their promoters for *WRINKLED1*, and this effort ended by finding several newly predicted targets in the upper glycolysis pathway and the pentose phosphate pathway, laying a foundation for future improvement of oil production in plants.

FAs in plants can be produced in two organelles, mitochondria, and plastids. In either of these organelles the synthesis of FAs requires lipoic acid (LA), a coenzyme essential for the activity of several key enzymes such as pyruvate dehydrogenase, 2-oxoglutarate dehydrogenase, and glycine decarboxylase. This coenzyme is synthesized by the concerted activities of octanoyltransferase and lipoyl synthase enzymes. The study described by Martins-Noguerol et al. characterized the effect of overexpressing these enzymes, cloned from the mitochondria of the seeds of *Helianthus annuus*, in *Arabidopsis thaliana*. Overexpression of these enzymes not only significantly altered some lipid species, mainly TGs and glycolipids, but also significantly affected levels of phosphatidylcholines and phosphatidylethanolamines in the transgenic Arabidopsis seeds, highlighting the importance of mitochondrial genes in the lipoylation pathway in seed metabolism.

Understanding the lipidomic profiles in seeds requires promising approaches to separate and identify different types of lipid metabolites. By using a liquid chromatography tandem mass spectrometry approach and comparing five different extraction methods, Romsdahl et al. took the challenge to improve lipid separation and quantification and to study the targeted FA compositions of non-polar and polar fractions in developing seeds and seedlings of pennycress. The analysis enlightened changes in composition and quantity of FAs that occurred during seed development, maturation and early germination. During seed development, for example, diacylglycerols predominantly contained long-chain FAs, contrasting with the very long chains FAs (VLCFAs) in mature seeds. The lack of VLCFAs during germination indicated that they are preferentially used for energy production at this stage.

The complexity of oil metabolism and the contribution of unconventional pathways to oil biosynthesis in seeds is reviewed by Sagun et al. This review highlighted the sources of carbon precursors and reductants for FA synthesis by using isolated plastids. The authors also referred to the importance of ^13^C-metabolic flux analysis as a tool to uncover these pathways in developing embryos. A list of key genes and regulators targeted to enhance oil yield and change the FAs composition in seeds is given. The list includes genes encoding transcription factors and genes related to boost the synthesis of FAs (“push”), increase TG assembly (“pull”), improve the storage of FAs into lipid droplets (“package”), and prevent the degradation of stored lipids (“protect”). The review suggests additional possible targets in the metabolic pathways that can be used to achieve desirable oil required for the industry, renewable biofuels, foods, and feeds.

In addition to FAs, seeds are an important source of essential amino acids. One of the most nutritional limiting amino acids is methionine. The study of Hacham et al. described the role of methionine γ-lyase (MGL), one of the central catabolic enzymes of methionine, in responses to abiotic stresses. The activity of this enzyme increases in developing seeds when the plants encounter heat and osmotic stress, leading to the reduction of the methionine level and the increase of another essential amino acid, isoleucine. Isoleucine is known to play a vital role as an osmoprotectant in stress adaptation and maintains the energy state of plants undergoing stress (Hildebrandt et al., 2015). Seeds of the MGL::RNAi lines showed the vital role of MGL in maintaining the seeds’ ability to germinate when exposed to stresses.

## Author contributions

RA: Writing – original draft, Writing – review & editing. EF: Writing – review & editing. JS: Writing – review & editing. APA: Writing – review & editing.
